# Supplementary material for: Are carbon emissions trading and green financial instruments synergistic? -Comprehensive quantitative research based on content analysis
Source: PLoS One. 2024 Mar 7;19(3):e0298601. doi: 10.1371/journal.pone.0298601 (PMC10919601; doi:10.1371/journal.pone.0298601)
Supplement: S1 Data — (ZIP) [file pone.0298601.s001.zip › Data/地级市Z_F工作报告.zip!/╣≤╓▌/╫±╥σ╩╨/└┤╫╘╣1⁄2╓┌║┼í╛┬φ┐╦╩2╛▌═°í┐_2014.docx]

各位代表：

　　现在，我代表市人民政府向大会作工作报告，请予审议，并请列席人员提出意见。

2013年工作回顾

过去一年，在市委的正确领导下，在市人大、市政协的监督和支持下，我们认真贯彻落实中央、省委省政府和市委各项决策部署，顶住宏观经济整体下行压力，克服持续高温干旱灾害等不利因素影响，凝心聚力，排难前进，千方百计稳增长、扩投资、调结构、增活力、惠民生，奋力保持了全市经济稳步发展、社会事业全面进步的良好势头。

一年来，我们保持经济较快增长，综合实力稳步提升。完成地区生产总值1584.7亿元，同比增长(下同)14%；50万元以上固定资产投资1566.1亿元，增长33.1%；财政总收入356.6亿元，增长23.7%；公共财政预算收入136.8亿元，增长21.8%；公共财政预算支出365.8亿元，增长14.3%；社会消费品零售总额470.4亿元，增长14.8%；城镇居民人均可支配收入20504元；农民人均纯收入6849元，增长13%；金融机构存、贷款余额分别完成2222.6亿元、1203.6亿元，均比年初增长29.8%；全面小康实现程度提高2个百分点、达78%；23.3万人脱贫，正安县和20个重点乡镇“减贫摘帽”。

一年来，我们着力优化经济结构，发展方式加快转变。三次产业结构由13.3︰46︰40.7调整为13.1︰47︰39.9。工业经济、民营经济、县域经济占生产总值比重分别为40.1%、52%、72.4%。工业经济加快发展。完成500万元以上工业企业增加值700亿元，增长17.5%；2000万元以上规模工业增加值677亿元，增长13.5%；企业利润总额占全省60%以上，扩大了在全省领先优势。实施产业园区成长工程和项目建设投产达产百日攻坚，完成工业投资724亿元，一批重点工业项目竣工投产，新增规模工业企业110户。特色优势产业快速发展，轻工化趋势明显，白酒、能源、材料、装备制造等主导产业增加值占规模工业的98%以上，轻重工业比为3︰1。主要工业产品产量稳定增长，其中完成白酒产量40万千升、煤炭产量1100万吨。淘汰落后产能78万吨，节能减排实现省下达目标。现代农业稳步发展。完成一产增加值207.9亿元，增长6.3%。农业基础不断夯实，主要农作物良种补贴实现全覆盖，实现粮食总产量259.8万吨。设施农业、现代种业、绿色有机农业和农产品加工业加快发展，规模以上农产品加工企业增至250户。“8531”工程成效明显，种植蔬菜、辣椒、高粱570多万亩，新增茶叶、中药材、干鲜果和改造竹林近60万亩，收购烟叶197.8万担，现有种养大户6万户、家庭农场150户，市级以上龙头企业达330家，农民专业合作社达2152个。第三产业提速发展。完成三产增加值632.7亿元，增长14.5%。接待游客4302万人次，旅游总收入359亿元，分别增长28.3%、28.2%。公路货物周转量和旅客周转量分别增长35%、32.6%。邮政、电信业务总量分别增长12.7%、15.8%。“引金入遵”工程深入实施，引进中信、兴业、中银富登等金融机构，银行、保险机构分别增至32家、20家。新雪域冷链物流、遵义国际商贸城、中国茶城等一批综合市场开业。改造中心城区农贸市场20个。“阿里巴巴·遵义产业带”建成运营，为300多户中小企业提供电商服务。

一年来，我们全力建设“三宜城市”，城市形象明显提升。城镇建设扎实推进。完成城镇建设投资900亿元。城镇化率提高到41%。新蒲新区项目入驻与功能完善齐头并进，“四横四纵”主干路网和湿地公园等一批标志性工程建成投用，中建·幸福城等一批重大项目入驻并加快推进，中心城区城市拓展框架基本成形。县城倍增计划初见成效，保利·未来城等城市综合体、茅台等小城镇加快建设。商品房施工面积2715.、竣工241.，建成保障性安居工程3.8万套。人居环境不断改善。狠抓生态文明建设和环境治理，中心城区空气优良率98.9%，县城以上饮用水质达标率100%，城镇建成区绿化率34.42%。列入国家低碳城市、新能源示范城市创建试点。赤水河流域环境保护加强。人工造林45.4万亩，治理石漠化面积160.4平方公里，森林覆盖率达51.24%。“四在农家·美丽乡村”创建14.5万户。“国家卫生城市”创建成果持续巩固。新增35个国家级生态镇和13个省级生态镇。凤冈、正安获“全国生态文明先进县”称号。习水获“国家卫生县城”称号。就业水平不断提升。新增城镇就业9.2万人、创业实体2.9万户，转移农村富余劳动力10.4万人，城镇登记失业率为2.96%。扶持微型企业2620户，带动就业1.55万人。民营经济市场主体达24万户，实现三年倍增。旅游基础不断完善。申报级景区1个，新增级景区4个、级景区3个，8家星级酒店试营业。荣获“中国最佳旅游投资城市奖”。赤水入选“2013亚热带地区最美热区生态城市”。

一年来，我们大力夯实基础设施，发展条件持续改善。综合交通运输体系初步形成。遵义机场开通航线12条，全年旅客吞吐量突破30万人（次），提前7年实现规划目标。仁怀机场、渝黔快速铁路全面开工。杭瑞、青檬、仁赤高速公路建成通车，新增高速公路通车里程，占全省新增里程的47.7%；务正、道安等高速公路加快建设；在国家和省路网规划调整中新增5条国道、12条省道；建成通村油路、乡镇客运站8个；全市公路通车里程达2.。构皮滩翻坝运输系统、乌江三级航道扩能、赤水河航道建设等加快推进，全市水域通航。水利设施不断夯实。中桥等3座中型水库主体完工，铜灌口等5座中小型水库成功截流，金龙等7座中小型水库开工建设。中心城区应急供水工程建成投用。治理病险水库63座，提前2年实现“十二五”规划目标。完成中小河流治理工程21个、烟水配套工程45个。新建微型水利工程8500处。治理水土流失面积240平方公里。解决了80.5万农村人口饮水安全问题。新增、恢复、改善灌溉面积35万亩。电力通讯基础更加完善。桐梓电厂1台机组建成发电，习水二郎电厂和一批小水电站加快建设。500千伏绥阳诗乡输变电等项目建成投用，新增变电容量200万千伏安。“智慧城市”建设加快。网络实现乡乡通。完成广播电视“村村通”10.4万户、“户户通”27.7万户。

一年来，我们深入推进改革开放，发展活力不断增强。在全省率先开展公共资源交易改革，完成交易额305亿元，节约和增值3.5亿元。省以下分税制财政体制改革、“营改增”改革顺利实施。以公立医院为重点的医药卫生体制改革加快推进。事业单位分类改革、城市基层管理体制改革、省直管仁怀试点等稳步实施。遵义演艺集团挂牌成立。水务一体化改革试点深入开展。组建了市铁投、能矿、水务等企业集团。上海对口帮扶合作全面展开。与中建、中铁等央企合作加强。19个产业园区、19个农业园区、21个城市综合体、17个旅游景区、12个小城镇纳入全省“5个100工程”。成功举办遵义（香港）招商旅游、上海优强企业助推遵义发展等重大招商活动。引进省外到位资金1165亿元，增长63.6%。实际利用外资1亿美元，增长30%。进出口总额3.8亿美元，增长22.5%。加快“人才创业和科技创新优选地”建设，遵义人才城建设启动，引进紧缺人才1740名。新增院士工作站3个，获省级以上科技项目240项，专利申请2400件，列入国家创新型试点城市。

一年来，我们努力构建和谐社会，人民生活日益改善。“十件民生实事”完成投资80亿元。社会事业加快发展。教育“9+计划深入实施，新建或改扩建乡镇公办幼儿园64所，遵义四中新蒲校区建成投用，中职学校全面免除学费，义务教育巩固率达88.2%，高中阶段毛入学率达75%以上；高等教育加速发展，遵义大学组建全面展开，遵义大学城建设加快推进，遵义医学院新蒲校区投入使用；民办教育健康发展。成功创建全国首批“国家公共文化服务体系示范区”；海龙屯列入世界文化遗产申报名单。人口出生率、自增率分别为10.37‰、4.99‰。基本药物制度、基本医疗保障制度、基本公共卫生服务有效实施，农村重大疾病保障病种扩大到24种；建成市中医院住院大楼和9家县级医院、75所乡镇卫生院。食品药品安全、卫生监督、疾病防控、爱国卫生、妇幼保健、血液安全等工作全面加强。社会管理不断创新。突发公共事件应急处置体系更加完善。安全生产事故起数和死亡人数实现“双降”，煤矿生产实现零死亡。开通信访直通车和网民留言交流平台。开展“法治遵义”、“平安遵义”创建活动，建成“天网工程”和“智能交通”平台，连续五次获“全国综治优秀平安城市”称号，第三次获全国综治“长安杯”。外事侨务、人防战备、国防教育、国家安全等工作有效开展。社会保障水平稳步提升。按照不低于10%的增幅提高城乡居民最低生活保障标准，城镇低保人均月增36元，农村低保人均年增189元。基本医疗保险参保127.9万人，新型农村合作医疗参合率98%以上。城乡居民养老保险参保280万人。城镇退休职工养老金人均月增171元。扶贫生态移民搬迁1.87万人，完成农村危房改造3.66万户。建成农村敬老院43所，五保对象集中供养率突破30%。与此同时，民族宗教、统计调查、水文气象、移民安置、档案、地方志、残疾人、红十字等工作有效推进。

一年来，我们扎实开展作风建设，政府效能不断提升。狠抓制度建设、“四风”整治和责任落实，勤政廉政建设取得新成效。修订完善了政府常务会议制度等议事规则。定期向市委、市人大报告和向市政协通报工作情况。181件人大代表建议和382件政协委员提案全部办结，满意率和基本满意率达100%。严格执行中央和省、市改进作风系列规定，累计暗访督查单位2000多个（次），查处违规人员1036人，督促整改问题900多个。网上行政审批和电子监察系统建设加快推进，电子政务外网认证系统试运行。40个市直单位实现财政预决算和“三公”经费公开。市级行政许可压缩30%，下放县级行政许可42项、下放率47%，清理保留非行政许可13项。强化国有企业和政府投资项目监督。审计监督、行政监察和行政问责力度不断加大。

各位代表！过去一年，面对前所未有的各方压力，我们坚定发展战略不动摇，紧紧依靠全市各族人民，万众一心，不争论、不折腾，大力实施主基调主战略，深入开展“五个梳理”，巩固了安定、团结、发展的良好局面。面对复杂严峻的经济形势，我们坚定发展目标不懈怠，积极应对，主动作为，采取领导挂帮、分类调度、季度考评、现场推进、开展“十大课题”研究等系列措施，促使经济逐月提升、逐季追赶，避免了大起大落，保持了稳中有进、稳中向好的发展态势。面对持续的高温干旱灾害，我们坚定民生为本不放松，广泛动员各方力量，众志成城抗大旱、保民生、促增收，夺取了抗旱救灾的重大胜利。面对增比进位的激烈竞争，我们坚定争先创优不气馁，保持和扩大工业、农业在全省的领先地位，加快城市建设和第三产业发展，稳步提升综合经济实力，并先后召开第二届旅发大会、全市工业发展大会和3次全市重点项目建设现场观摩会，承办全省第三次项目观摩会、2013环中国国际公路自行车赛等重大活动，充分展示了名城遵义的发展成果和良好形象。

这些成绩的取得，是省委、省政府和市委正确领导的结果，是市人大、市政协监督支持的结果，是全市广大干部群众团结奋斗和各方大力支持的结果！在此，我代表市人民政府，向全市各族人民，向人大代表、政协委员，向各民主党派、工商联、无党派人士、人民团体，向驻遵人民解放军指战员、民兵预备役人员、武警消防官兵和人民警察，向历届老领导老同志，向所有关心支持遵义发展的各界人士，表示衷心的感谢！

在充分肯定成绩的同时，我们也清醒地看到存在的问题。主要是：经济持续快速增长的动力不足，发展速度放缓，综合排位下滑；转型升级压力大，部分企业生产经营困难；区域发展不平衡，城乡二元结构突出；农业抗灾能力弱，农民增收渠道窄；财政收支矛盾突出，融资和用地瓶颈明显；市场化体制机制滞后，发展环境有待改善；政府工作有待进一步改进，有的部门执行力落实力不强、“庸懒慢浮贪”现象不同程度存在。对此，我们将以强烈的责任感和紧迫感，采取更加有力有效的措施加以解决。

2014年工作目标和主要任务

今年是全面深化改革之年，也是实现“十二五”规划目标的攻坚之年。我市正处于发展加速期、结构调整期、改革攻坚期，面临着宏观政策取向利好、市场预期向好、自身发展条件改善等有利形势。特别是随着交通等基础设施的不断完善，产业基础不断巩固，发展平台加快形成，改革等红利集中释放，开启了科学发展、加速发展、跨越发展的新征程。面对新机遇与新挑战，我们必须牢固树立进取意识、机遇意识、责任意识，以更加解放的思想和巨大的政治勇气，坚持和完善发展思路；以更加开阔的视野和坚定的信念，大力推进提速转型；以更加务实的作风和勤政为民的理念，全力保障和改善民生，务求各项工作取得新突破，开创全市发展新局面。

今年政府工作的总体要求是：认真贯彻落实党的十八大、十八届三中全会、省委十一届四次全会、市委四届四次全会和中央、省、市经济工作会议精神，坚持稳中求进、提速转型、又好又快的总基调，坚持主题主线和主基调主战略，充分发挥市场在资源配置中的决定性作用，把改革创新、“五个梳理”贯穿于经济社会发展各领域，决战“十二五”，打好攻坚战，以实施“八大工程”为抓手，全力打造遵义发展升级版。以更加解放的思想、更加开阔的思路、更加扎实的作风、更加进取的精神，统筹推进全市经济、政治、文化、社会和生态文明建设，为建设“五个遵义”，在全省率先建成全面小康社会、率先向现代化迈进打下坚实的基础。

全年经济社会发展的主要预期目标是：完成地区生产总值1910亿元，增长15%以上，力争达到17%；三次产业结构调整为11︰46.1︰42.9；500万元以上工业企业增加值860亿元，增长21%以上；50万元以上固定资产投资2300亿元，增长35%以上；财政总收入435亿元、公共财政预算收入163亿元，分别增长22%、20%；社会消费品零售总额556亿元，增长17%以上；城镇居民人均可支配收入23785元，增长16%以上；农民人均纯收入8013元，增长17%以上；居民消费价格指数、单位生产总值能耗和地耗、主要污染物排放总量控制在省下达指标以内；全面小康实现程度提高4个百分点。

围绕以上目标任务，我们将着力抓好七个方面工作：

一、着力全面深化改革，增强发展活力

改革是最大的红利。我们要切实用好改革关键一招，以务求必胜的信心、敢为人先的勇气，大力深化各项改革。今年要重点抓好十项改革：深化行政管理体制改革。推进行政审批制度改革，简化办事程序，提高服务效能。加快转变政府职能，完成市、县政府机构改革。推进事业单位法人治理结构建设试点。严控“三公”经费支出，继续压缩5%的行政经费用于实施教育“9+3”计划。制定政府向社会购买公共服务的指导意见。积极申报较大的市。力争实现遵义县撤县设区，积极推动绥阳撤县设区和桐梓、湄潭撤县设市，推进撤乡建镇和镇改街道办。深化投资领域改革。落实企业投资主体地位，对申请国家和省级补助资金的企业投资项目，除国家和省有明确要求外，市级不再审批。鼓励社会资本参与社会公共服务、城市基础设施和新农村建设。深化招投标和项目监管制度改革。加强公共资源交易管理，政府投资和使用国有资金在法定限额以上的工程项目、政府采购、产权交易、土地使用权出让等，进入公共资源交易中心交易。健全重大建设项目稽查制度，建立公众参与工程项目监督机制。深化统筹城乡综合配套改革。改革完善农村宅基地制度，建立“政府引导、市场调节、农民自愿、依法有偿”的土地流转机制，推广湄潭农村土地制度改革和凤冈“三资转换”试验成果，建立农村产权流转交易市场。深化户籍制度改革。放开落户限制，建立财政转移支付同农业转移人口市民化挂钩机制，推进城镇基本公共服务常住人口全覆盖，积极稳妥推进农业转移人口市民化。深化民营经济体制改革。全面清理阻碍经济公平发展的条条框框，制定民营企业进入特许经营领域具体办法，鼓励、支持民营经济参与重组国有企业，大力发展混合所有制经济。深化资源配置体制改革。推行优势矿种矿业权招拍挂出让制度。启动煤炭资源税从价计征改革。深化财税体制改革。完善分税制财政管理体制，扩大“营改增”试点。启动编制国有资本经营预算，建立国有资本收益上缴公共财政管理机制。出台市级国有资产运营管理指导意见，制定国有投资公司绩效考评制度。深化社会事业领域改革。全面落实教育、就业、社保、卫生计生和食品药品监管等领域改革任务。推行县域内校长教师交流轮岗制度。组建遵义体育产业发展公司。实施单独两孩生育政策。实现行业协会与行政机关彻底脱钩。深化电网建设改革。推进自备电厂和大用户直供电试点，争取电力局域网建设取得新进展，推动煤电铝、煤电化等一体化发展。

二、着力优化产业结构，打造遵义经济升级版

加快工业转型升级。坚持走新型工业化道路，实施工业转型升级三年行动计划。大力推进省级经开区和产业园区建设，完善园区产业规划，理顺管理体制；将35%以上的新增建设用地用于产业园区，推进基础设施建设攻坚，加快水电路、标准厂房、污水处理等设施建设，新建标准厂房100万平方米以上，完成园区投资600亿元以上。加快特色轻工、装备制造、新型材料、能源、化工、战略性新兴产业等六大产业发展。推进“中国酱香·赤水河谷”品牌示范区建设，加快茅台酒、习酒等企业技改扩能项目建设，引导企业拓展市场，提高市场占有率，实现白酒产量50万千升以上。加快煤炭、页岩气、风能等资源勘探和开发，完成煤矿兼并重组，实现煤炭产量1300万吨以上。加快电子信息、生物制药、新型合金材料等高新产业发展，培育引进一批高新技术企业，高新技术企业达到55家。促进科技与经济紧密结合、信息化和工业化深度融合，发展移动互联网和物联网产业，培育一批“两化融合”示范企业和示范园区。开工建设务川氧化铝和天义厂、061基地整体搬迁等项目，推进遵义卷烟厂、湄潭复烤厂、中心城区重点企业异地技改，完成航天成功汽车、贵州钢绳股份有限公司技改一期等项目建设，建成投产项目150个以上，新增规模以上工业企业100家以上，工业经济比重提高1.2个百分点以上。

加快服务业发展。坚持以文化旅游业为龙头，实施服务业培育壮大三年行动计划，健全现代服务业体系。大力发展文化旅游业。巩固提升“国家公共文化服务体系示范区”创建成果，健全现代文化市场体系，加快市县文化馆和县级图书馆、体育场馆、档案馆建设，加强非物质文化挖掘、传承和保护工作，筹建遵义人民出版社、市文物保护中心和“三线建设”文化产业园，全力推进海龙屯世界文化遗产申报工作。坚持全景域理念，高标准、高品质促进旅游业发展。办好第三届旅发大会。加快文化旅游发展创新区和重点旅游景区建设，完成遵义会议陈列馆改扩建、长征文化博览园（一期）等项目建设，力争赤水大瀑布级景区申报成功。大力发展生态旅游、少数民族文化旅游。加快星级酒店、乡村旅馆、旅游地产等建设和旅游商品开发，建成星长征、茅台迎宾馆等高星级酒店和遵义会议会展中心，中心城区外的其他县（市）至少各建成投用1家四星级以上酒店。旅游接待人次和总收入分别增长20%、22%以上。大力发展生产性服务业。加快交通运输、仓储物流、营销贸易、电子商务发展，推进南宫山商贸物流中心、阁老坝和李家湾铁路物流中心等项目建设，做强“阿里巴巴·遵义产业带”电商平台，推进“新网工程”、“万村千乡市场工程”建设，新建100个村级综合服务站和10个区域配送中心。继续实施“引金入遵”工程，启动新蒲金融商务中心建设，组建农村商业银行，推动村镇银行县域全覆盖，加快小额贷款、融资担保等地方中小型金融企业健康发展。扶持文化会展、规划设计、中介服务等产业发展，引进具有国际性、全国性、区域性影响的企业，发展总部经济。大力发展生活性服务业。改造提升中心城区商业功能，引导发展健康餐饮、商务娱乐、休闲养生等行业，推进奥特莱斯商业广场、月星国际城等项目建设，打造特色餐饮美食街15条以上。优化城市拓展区商业功能规划布局，推动餐饮、住宿、家政等行业健康发展。加快生鲜商品流通追溯体系和社区便民直营店试点项目建设。

加快现代农业发展。稳定粮食生产，实施农业特色产业效益提升三年行动计划，创新农业生产经营方式，大力实施“1234”战略，深入推进“8531”和“321”工程，加快传统农业向现代农业、农业大市向农业强市、黔北粮仓向黔北钱仓“三个转变”。提高良种茶、精品果、特色药材、优质商品蔬菜等经济作物比重，实现粮食产量280万吨以上、肉类产量58万吨，收购烟叶170万担，推进30万吨粮油仓储建设。加快农村土地规模化集约化经营，通过土地流转、招商引资等方式，大力培育专业大户、家庭农场、休闲农庄等新型农业经营主体。推进无公害、绿色、有机产品和地理标志认证，支持创建特色农产品品牌和商标注册，打造100个市级以上农产品品牌，做强一批市级以上龙头企业。抓好国家新增千亿斤粮食生产能力建设和农业综合开发项目，推进土地整治和中低产田土改造，加强高标准农田建设，健全农产品质量安全监测体系，加快农业机械化、信息化进程，加强水文气象监测预报预警，完善农业防灾减灾体系，夯实农业基础。加快发展基层供销合作社、村级综合服务站和农村合作经济组织，构建“三农”服务综合平台。

三、着力统筹城乡建设，打造城乡发展升级版

统筹“四大区域”发展。按照“中部率先崛起、西部全域突破、东部联动开发、北部全速攻坚”要求，突出分类指导，强化分工协作，坚定不移推进“四大区域”发展。编制完成分区域总体规划和空间布局规划，分区域制定发展指标和考核体系，推动“四大区域”各展所长、竞相发展，不断增强县域经济综合实力，县域经济比重提高1个百分点以上。坚持“以村保镇、以镇保县、以县保市”，扎实开展以县为单位的全面小康创建活动，重点抓好4个示范县、14个示范镇、35个示范村创建工作，在各县（市、区）建成1-2个示范镇、示范村。

推进新型城镇化。坚持产城互动、城乡一体，推动人的城镇化，构建城镇化发展新格局，城镇化率提高2个百分点以上。注重规划的连续性与严肃性，优化城市发展规划，加快中心城区建设，主动融入黔中城市群发展，启动汇川至绥阳、新蒲至绥阳、湄潭至新蒲等城际快速通道建设，完善新蒲新区、南部新城、北部新城骨干路网和市政设施，推进新蒲新区“十大标志性工程”等重点项目建设。深入实施县城倍增计划，启动“黔北城市群”规划，推进城市综合体建设，不断完善城市功能，提升承载能力。加快“六型”示范小城镇建设，优化乡镇总体规划和控制性详规，完善水电路讯、教育卫生和垃圾污水处理等公共设施，建设10个以上特色旅游小城镇。提高规划编制管理和城市管理水平，探索城市管理网格化、规范化机制，推进“智慧城市”、“公交都市”建设，切实整治中心城区道路交通拥堵问题。整合各类政策资源，依法开展征收拆迁补偿安置工作，严厉打击“两违”行为。

改善农村条件。实施“四在农家·美丽乡村”基础设施六项行动计划和景观通道建设，保护自然景观和村寨特色，拓展和提升“四在农家”内涵，打造33个精品示范点。实施10.5万亩植树造林和景观绿化工程。实施通村油（水泥）路建设，改造县、乡道和21座危桥。加快农村文娱设施、医疗设施建设，建成42所中心乡镇卫生院、397间村卫生室。新建或改扩建农村敬老院65所，实现农村敬老院全覆盖。

实施“八大工程”。按照“全体动员、集中力量、市县联动、分级负责、分类实施”要求，大力实施中心城区城市道路交通改善、环保模范城市创建、文化旅游景区精品、旅游服务能力提升、“四在农家·美丽乡村”升级、教育医疗民生建设、平安和谐、行政效能提升“八大工程”，推动城乡面貌大变样，提升遵义知名度和美誉度。建立领导挂帮、分级调度机制，做到精细化、精准化、科学化施工，实施项目169个，完成投资300亿元以上。

四、着力扩大投资，突破重点项目建设

全面优化投资结构。推进“5个100工程”建设，充分发挥投资主阵地和主平台作用。发挥市场机制作用，多渠道加大特色产业、基础设施、民生工程等投资，完成工业投资920亿元、城建投资1200亿元、交通投资220亿元、水利投资48亿元、旅游和市场物流投资110亿元、农村基础设施和社会事业投资145亿元。挖掘民间投资潜力，完成非公有制经济投资1000亿元左右。

加快基础设施建设。加速完善现代综合交通运输体系。启动遵义机场站坪扩建工程，力争开通至大连、郑州、青岛、南京、南宁、桂林航线，积极争取开通国际航线航班；加快仁怀机场建设和黔北机场前期工作。加快渝黔快速铁路建设，开工建设川黔铁路外迁东移工程，启动攀昭黔铁路(遵义段)、遵义至泸州铁路和贵阳至遵义城际铁路、中心城区轨道交通前期工作。深入开展交通基础设施建设“三年会战”，建成余凯、檬乐、务正等高速公路，新增通车里程；加快道安、江安等高速公路建设；新开工贵遵复线、江津经习水至古蔺、正安至习水、务川至德江等高速公路；加快遵义至仁怀复线、绥阳至正安等高速公路前期工作；改造国、省道以上。加快赤水河上游航道整治及乌江渡库区、桐梓河等航道建设，新建渡口20个。加快水利设施建设。实施水利“三大会战”，建成中桥水库；实现新桥、大沙河等5座水库下闸蓄水，新增年供水能力1.；加快麻沟、大沙坝等15座水库建设；开工建设上水、水源坝等7个骨干水源工程和27个中小河流水文监测站。开工建设8个烟草水源工程建设。实施中小河流治理项目23个，新增、恢复、改善灌溉面积16.8万亩以上。加快电力通讯设施建设。全面建成桐梓电厂和牛都、沙阡水电站，加快二郎电厂和肖家湾、牛角塘等水电站建设，开工建设高生、角木塘等水电站。完成500千伏习水至重庆输变电、220千伏桐梓夜郎输变电等工程，新建及改造220千伏线路、110千伏线路、35千伏线路，新增变电容量202万千伏安。加快“三网融合”、“宽带中国”进程。建成网络基站5000个，实现乡镇以上区域和高速公路沿线、旅游景点全覆盖。建成市政务云计算中心。推进农村数字电视无线覆盖工程。

突破项目建设瓶颈。简化审批程序，推行并联审批，建立重大项目专人全程跟踪服务机制。加大市、县土地收储力度，加快投融资平台建设，推进投融资平台资源整合，加强政银企对接合作，全年融资1000亿元以上。积极推动企业上市融资。建立项目建设推进长效机制，分区域、按季度开展项目观摩，实行重大项目建设进度通报和排名制。全年实施650个省市重大工程，新开工262个、竣工74个。

五、着力扩大开放，构筑开放型经济高地

深化区域合作。坚持借力上海、融入成渝，加强与经济发达地区合作。抢抓上海对口帮扶机遇，加强双方产业、教育、人才、技术、资金等合作，引进一批电子信息、整机装备、生物制药等优强企业。建立产业园区合作共建机制，按照一区多园发展战略，高起点、高标准推进遵义国家经开区东区（新蒲）上海产业园、遵义国家经开区与漕河泾共建产业园建设。加强与集团企业、金融机构、高校、科研院所的合作开发。申建遵义综合保税区，推进国家级外贸转型升级示范基地建设。实施外贸翻番计划，进出口总额增长20%以上、实际利用外资增长30%以上。

加强招商引资。扎实开展“招商引资年”活动，完善招商引资激励、工作考核和投资环境评价机制，落实招商引资主体责任。加强招商引资项目储备，依托战略、政策、市场、技术谋划一批重大招商引资项目。创新工作机制，推行小分队招商、以商招商、中介招商、定点招商，着力招大引强。健全投资服务体系，推行招商项目全程代办，强化跟踪服务，营造亲商、扶商、安商氛围。引进省外到位资金1500亿元以上、增长30%，招商引资项目履约率达90%以上，资金到位率、投产达产率分别提高5个百分点以上。

壮大民营经济。启动新一轮民营经济倍增计划，实施中小企业成长工程，建成市级中小企业服务中心，健全中小企业信用担保体系，鼓励中小企业进园区和标准厂房，建设民营经济示范园区。引导中小企业围绕大企业协作配套，大力发展配套型、劳动密集型中小企业。新增民营经济主体10万户，民营经济比重提高3个百分点。扶持微型企业2800户，带动就业1.4万人以上。

六、着力生态环境建设，促进可持续发展

加强生态文明建设。守住生态底线，抓好水利、生态、石漠化治理“三位一体”规划，治理石漠化面积170平方公里、水土流失面积300平方公里。开展赤水河流域生态保护红线划定、自然资源登记确权、生态补偿、水资源有偿使用、环境污染第三方治理及“河长制”考核等改革试点，启动赤水河“四河四带”规划建设。加快赤水河流域污水、生活垃圾处理全覆盖。力争三县三镇两村成功创建国家级生态名片、一县两镇三村成功创建省级生态名片。完成营造林75万亩，森林覆盖率达51.8%。

加强环境保护治理。全力实施“国家环保模范城市”创建攻坚，确保创建成功。加强饮用水源、城区空气质量监测和治理。完成中心城区生活垃圾和污泥利用水泥窑协同处置项目及垃圾中转库建设、海龙和金鼎山污水处理、高坪和高泥等河流截污治理，启动中心城区南部污水处理厂二期、中水回用及蓄水工程建设。狠抓节能减排，推行清洁生产，深入开展循环经济示范创建和国家低碳城市试点、国家新能源示范城市创建，加大酒糟、赤泥等工业固体废弃物综合利用，提高工业用水循环利用率，积极防治工业和生活污染。完成节能减排目标，淘汰落后产能117万吨。

加强耕地保护。严守耕地红线，注重集约节约用地，完成土地利用总体规划中期评估，保护1000亩以上连片（大坝）耕地。建立城乡统一的建设用地市场，在符合规划和用地管制情况下，推行农村集体经营性建设用地出让、租赁、入股，实行与国有土地同等入市、同权同价。继续实施增减挂钩，严格实行耕地占补平衡。

七、着力保障和改善民生，推动社会和谐

统筹社会事业发展。深入实施教育“9+3”计划，加快城乡学校标准化和信息化建设，促进义务教育均衡发展，义务教育巩固率达88.5%以上，高中阶段毛入学率达80%以上；建立高职高专办学经费保障机制，推广职业教育进园区模式，构建现代职业教育体系；加快遵义大学城建设和遵义大学组建，支持遵义医学院申建遵义医科大学；推进学前教育、民办教育、特殊教育健康协调发展。开展全国创新型城市创建，争取省级以上科研项目260项，专利申请2600件。深入实施“百千万人才计划”和“15851”人才精英工程，加快人才服务体系和遵义人才城、人才网建设，引进一批急需紧缺人才。推进基层卫计整合，实施国家免费孕前优生健康检查，加大出生人口性别比综合治理，推动流动人口计生基本公共服务均等化，人口出生率、自增率分别控制在10.55‰、5.05‰内。提高市级医疗服务能力，完善县、乡、村医疗服务体系，推进城乡医保一体化，实现新农合参合率98%以上。巩固提升“创卫”成果。加快疾病防控、卫生监督、中心血站等综合服务项目建设。健全市、县、乡、村食品药品监管体系。加强妇幼保健等工作。完成第三次全国经济普查和第九届村（居）民委员会换届选举。深入开展民族团结进步繁荣发展示范区建设和宗教“和谐寺观教堂”创建活动。加强外事侨务、人民防空、国防教育、机构编制、档案、双拥、慈善、移民、地方志、残疾人、红十字等工作。

健全社会保障体系。实施更加积极的就业政策，完善城乡均等公共就业服务体系，加大创业培训力度，抓好高校毕业生、就业困难群体和失地农民就业，新增城镇就业10.8万人，失业人员再就业1.8万人，转移农村富余劳动力7.5万人，城镇登记失业率控制在4.2%以内。健全社会保障体系，抓好“八大体系”建设，筑牢社会保障底线。完善城乡低保动态管理机制，提高城乡居民最低生活保障标准。统筹推进城镇保障房、扶贫生态移民房建设和农村危房改造，完成保障性安居工程5万套、扶贫生态移民搬迁1.6万人、农村危房改造3.6万户。加强城乡居民生活必需品价格监测，健全低收入群体价格临时补贴与物价上涨联动机制。

深入实施扶贫攻坚。健全扶贫开发工作责任制和精准扶贫工作机制，深化产业扶贫、集团帮扶、对口帮扶等政策措施，切实做到结对帮扶、产业扶持、教育培训、农村危改、扶贫生态移民、基础设施到村到户。加大扶贫投入，落实目标、任务、资金、权责“四到县”制度，推进扶贫攻坚示范区建设，切实改善贫困地区基本生产生活条件、拓宽基本增收门路、提高基本素质。实现务川自治县和全市20个重点乡镇“减贫摘帽”，减少农村贫困人口20万人以上。

加强和创新社会治理。牢固树立安全发展理念，强化安全生产责任，健全安全监管长效机制，加强隐患排查治理，健全风险控制和应急救援体系，坚决杜绝重特大事故，严格防止较大事故，有效预防一般事故。强化社区服务功能，深入开展“四民社区”创建活动。加强公民道德建设，培育和践行社会主义核心价值观。扎实推进“平安遵义”建设，深入开展“严打整治”专项行动，加强社会治安综合治理。大力推广矛盾纠纷排查化解“余庆经验”，创新社会矛盾排查化解机制，加强特殊人群管理，推进诉访分离，建立涉法涉诉信访依法终结制度，改进社会治理方式。深入推进“六五”普法，建立覆盖城乡的公共法律服务体系。

全力办好民生实事。坚持民生为先、先急后缓原则，重点解决一批人民群众最关心、最直接、最现实的利益问题。

各位代表！新的形势和新的任务对政府工作和自身建设提出了新的要求。打铁还需自身硬！我们必须严守政治纪律，始终与党中央保持高度一致，在市委的坚强领导下，将主基调主战略贯穿于经济社会发展全过程，落实于政府执行工作之中，深入开展党的群众路线教育实践活动，全面增强政府的决策力、执行力和公信力。我们必须大力解放思想，坚持“十破十立”，深入开展“五个梳理”，瞄准省内外先进地区对标，干字当头、敢字为先、拼字求胜，以高度的责任感、紧迫感和使命感，大胆突破常规，打破一切阻碍发展的惯性思维和习惯做法，用改革的思路、创新的办法、市场的机制和超常规的手段推动大发展、大跨越。我们必须严格依法行政，自觉接受市人大及其常委会的法律和工作监督，市政协的民主监督，认真办理人大代表、政协委员建议提案；高度重视民主党派、工商联和无党派人士的意见建议，充分发挥工会、共青团、妇联等人民团体的桥梁纽带作用；重视社会公众监督和舆论监督，建立公众直通交流平台；加强行政监察和审计监督；做好政策风险性评估；推行权力清单制度，健全政府法律顾问制度，完善重大决策合法性审查和规范性文件备案审查制度；深化行政执法体制改革，加强执法监督，规范自由裁量权。我们必须切实转变作风，认真落实中央、省委和市委关于作风建设的系列规定，开展“三晒两评一报告”监督评议活动，以铁的纪律整治机关作风，全面掀起“效能革命”；加快政务服务、公共资源交易和电子政务平台建设，推行一站式审批、一条龙服务，不断提高政务服务水平；大力弘扬钉钉子精神，建立目标倒逼机制，强化督促检查和行政问责，确保工作落实。我们必须加强廉政建设，严格落实党风廉政建设责任制，严格遵守廉政准则，加强惩治和预防腐败体系建设，以改革创新精神推进政府廉政建设和反腐败工作；强化廉政教育与行政监察，深化领导干部经济责任审计，积极探索资源资产责任审计；加强政府投资项目监管，加大对中介机构、行业协会的引导、评价和监督，严防工程建设、矿产开发、土地出让等领域腐败问题滋生；坚决纠正部门和行业不正之风，切实解决以权谋私、与民争利等损害国家和群众利益的问题，敬仰理想，敬畏职责，严于律己，大力营造风清气正的政务环境，以务实高效的作风服务于民，以勤政清廉的形象取信于民。

各位代表！梦想承载希望，奋斗开创未来。让我们紧密团结在以习近平同志为总书记的党中央周围，在省委、省政府和市委的坚强领导下，抢抓机遇，改革创新，克难攻坚，破浪前行，为打造遵义经济升级版，全面建设小康遵义、法治遵义、人文遵义、平安遵义、生态遵义而努力奋斗！
